# Perspective to precision medicine in scleroderma

**DOI:** 10.3389/fimmu.2023.1298665

**Published:** 2024-01-18

**Authors:** Kazuhiro Komura, Koichi Yanaba, Jean-David Bouaziz, Ayumi Yoshizaki, Minoru Hasegawa, John Varga, Kazuhiko Takehara, Takashi Matsushita

**Affiliations:** ^1^ Department of Dermatology, Kanazawa Red Cross Hospital, Japanese Red Cross Society, Kanazawa, Japan; ^2^ Northwestern Scleroderma Program, Division of Rheumatology, Northwestern University Feinberg School of Medicine, Chicago, IL, United States; ^3^ Department of Dermatology, Jikei University, Tokyo, Japan; ^4^ Department of Dermatology, Hôspital Saint-Louis, Paris, France; ^5^ Department of Dermatology, University of Tokyo, Tokyo, Japan; ^6^ Department of Dermatology, Fukui University, Fukui, Japan; ^7^ Department of Dermatology, Kanazawa University, Kanazawa, Japan

**Keywords:** systemic sclerosis, fibrosis, precision medicine, biomarkers, therapy

## Abstract

Systemic sclerosis (SSc) is a rare and heterogeneous disease with no relevant environmental trigger or significant responsible gene. It has been and will continue to be difficult to identify large enough patients to conduct classic population-based epidemiologic exposure/non-exposure studies with adequate power to ascertain environmental and genetic risk factors for these entities. The complexity of pathogenesis and heterogeneity are likely to require personalized/precision medicine for SSc. Since several potential drugs are currently available for specific patients if not whole SSc, classification of SSc seems to form the foundation for a better therapeutic strategy. To date, SSc has been classified based on the extent/severity of the affected area as well as some disease markers, including the autoantibody profile. However, such an analysis should also lead to improvements in the design of appropriately stratified clinical trials to determine the effects and prediction of targeted therapies. An approach based on drug response preclinically conducted using patients’ own fibroblasts *in vitro*, can provide a precise disease marker/therapeutic selection for clinical practice. Because scleroderma dermal fibroblasts have a persistent hyper-productive phenotype occurring not only in person, but also in cell culture conditions. Thus, an accumulating approach based on disease markers ensures progression and de-escalation to re-establish a better life with a personally optimized drug environment after the onset of SSc.

## 1 Introduction

Systemic sclerosis (SSc) or scleroderma is characterized by immune dysregulation, obliterative microvasculopathy, and fibrosis, which are significant causes of mortality occurring in the whole body (1–3). Fibrosis is caused by the excessive deposition of collagen and other extracellular matrix components produced by activated fibroblasts and α-smooth muscle actin-positive myofibroblasts. *In vitro*, scleroderma patient derived skin fibroblast synthesizes more collagen than fibroblasts from healthy humans (4–6). This *in vitro* evidence has suggested the role of epigenetics in the pathogenesis of SSc (7–11). Exogenous circumstances promote persistent cellular fibrotic phenotypes in fibroblasts, going over the endogenous predisposition in SSc patients (12–18).

Epigenetics describe heritable changes in gene expression that is independent on DNA sequence. Recent discoveries have linked to targeting DNA methylation, chromosome remodeling, and RNA turnover. Although epigenetic mechanisms help to protect cells from parasitic elements, this defense can complicate genetic manipulation. Essential to normal development, epigenetic controls are misdirected to human diseases (19). Certain environmental imprinting settings can be transmitted to future generations, although the role of these changes in the transmission of disease to humans remains unclear. The best-studied epigenetic modification of DNA is methylation of DNA by methyltransferases on cytosine residues of paired CG sequences and acetylation of lysine residues on histones by histone acetylases (20). For example, Oxidative DNA damage-mediated histone deacetylation activates the Wnt signaling pathway and fibrotic processes in SSc (21). Improved miR-150, one of miRNAs in SSc fibroblasts, normalized activated TGF beta signals and fibrosis (22). These epigenetic changes can explain why scleroderma dermal fibroblasts have a persistent hyper-productive phenotype expressed not only in person but also in cell culture conditions.

**Figure 1:**
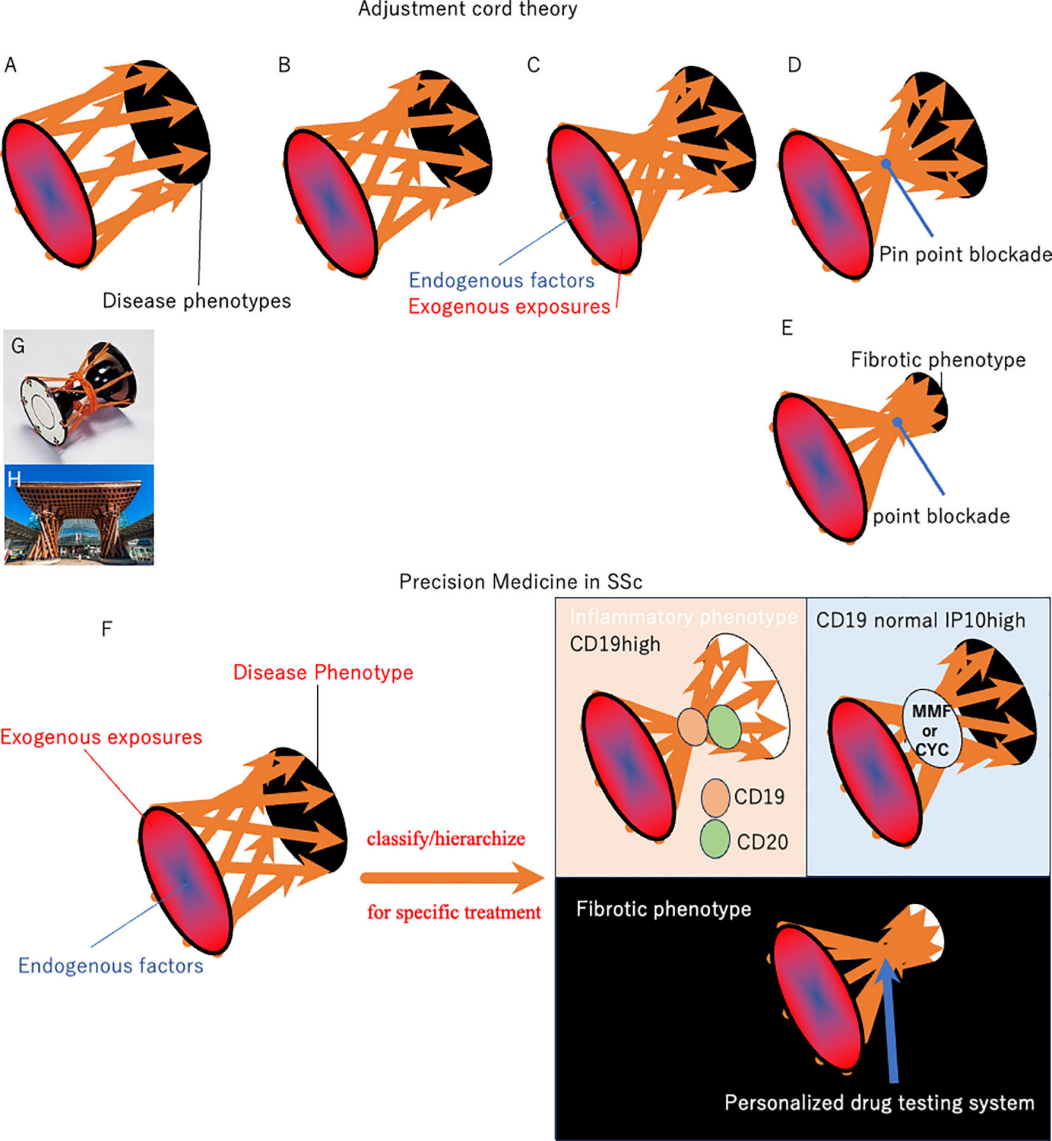
Schematic of pathological pathways in SSc. Various endogenous factors establish heterogeneous phenotypic expression in SSc in cooperation with exogenous triggers **(A)**. The pathogenetic stream may be narrowed weakly during the establishment of the phenotypic characteristics (panels **(B, C)**. The results from clinical practice indicate that antifibrotic drugs, including IM, Tocilizumab, are effective for specific SSc patients, although these were not validated in randomized trials in patients with SSc. This indicates that narrow-target therapy partially abrogates wide and complex pathways to SSc, while others bypass the blocking point, resulting in continuous development of SSc phenotypes. Considering the pathological pathway, the current approach using existing biomarkers and blocking therapy does not reach the focus point, resulting in incomplete efficacy of single-molecule blockers in SSc (panel **(B)**. In contrast, the pathogenesis of psoriasis and atopic dermatitis consists of umbrella-like upstream, umbrella-like downstream, and focus point: IL-23 and JAK1 are near the focus point in the pathogenic motif of psoriasis and atopic dermatitis, respectively (panel **(D)**. Alternatively, focusing on the final fibrotic process is empowered to change patient mortality or quality of life, since the final step for phenotypic fibrosis seems to be crucial for mortality and quality of life in most patients with SSc (panel **(E)**. Biomarker studies may classify/hierarchize patients with SSc to select an efficient patient-entry design in clinical trials (panel **(F)**. Further characterization of the key genomic and immune pathways involved will help identify novel disease mechanisms and therapeutic targets for precision medicine initiatives in SSc. The Tsuzumi column is the origin of the motif used in the pathological pathway described in this paper (panel **(G)**. Tsuzumi, a traditional Japanese drum played in Nou, closely correlated with the traditional culture of Kanazawa City in Japan. Many citizens were initially surprised at the modern entrance when they appeared in front of Kanazawa station in 2005 (panel **(H)**. The station’s wooden hand-drum-shaped Tsuzumi pillar and glass umbrella-shaped Motenashi Dome were controversial, because they are novel and new shapes staying in front of this traditional castle town: Kanazawa is one of the best-preserved Japanese cities after the WWII bombings. However, the station has become popular with tourists because of its beautiful sleek design. Tsuzumi changed the beat by adjusting the cord placed at the center. We hypothesize “Tsuzumi adjustment cord theory” hoping that many clinical trials tighten the center cord and discover the hub/focus point of SSc safely. Efficacy/safety and paradoxical reactions during targeting therapy trial is key findings for tightening the code to make two umbrellas and a focus point from Tuzumi pillar.

The clinical course of SSc varies substantially, and the pathogenesis and degree of fibroblast production depends on the individual burden of environmental exposure to pathogens and gene susceptibility (**Figure 1**). The response to antifibrotic drugs is also heterogeneous among patients, resulting in a lack of FDA-approved antifibrotic drugs for SSc from randomly controlled clinical trials with large number of patients (23–28).Traditional studies identifying anti-fibrotic drugs were conducted in optimized culture microenvironment and these *in vitro* studies have proposed several anti-fibrotic drugs, including Imatinib mesylate, c-Abl tyrosine kinase, and a line of PPARγ activators (23–28). However, genetics, epigenetics and environmental/exogenous affects phenotypic features of cell cultures and organism variation (29–31). In schematic pathological pathways of SSc, various endogenous factors establish heterogeneous phenotypic expression in cooperation with exogenous triggers. The pathogenetic spiral vectors may be narrowed weekly during the establishment of the phenotypic characteristics (**Figures 1B, C**). Within this motif, narrow portion could be a treatment target. However, existing anti-fibrotics are efficacious only in subpopulations of SSc patients, which were not sufficient to show differences in large-scale placebo-controlled studies in SSc (32, 33). This means some pathological vectors bypass the blocking by treatments.

## 2 Precision medicine

Until now most medical treatments were engineered for the “average patient.” As a result of this “one-size-fits-all” approach based on the recommendation from randomized controlled trials, treatments can be very successful for some patients, but not for others with heterogeneous diseases. Most immunological skin diseases, including SSc, do not have a standard therapy, since the biological pathophysiology and efficacy of the therapeutic intervention are highly variable between individual patients. Nonetheless, a sophisticated strategy improved therapeutic efficacy in Psoriasis, one of heterogenous immunological skin diseases. IL23 or IL17 blockade clears over 50% psoriasis patients if it continues (34–36). This indicates that IL23/IL17 aggregate complex pathological vectors in psoriasis (**Figure 1D**).

Precision medicine is an emerging approach for disease treatment and prevention that considers individual variability in genes and the environment for each person. Although some advances in precision medicine have been made, the practice is not currently in use for most diseases. Precision medicine can provide medical professionals with specific treatments for the correct patient group at the appropriate time (37, 38).

Excessive exposure to exogenous triggers causes disease when it exceeds the threshold limited by endogenous susceptibility. At that time, there is an opportunity to mitigate the crisis by optimally regulating external and internal exposure through circumstances and treatment. Appropriate disease markers early in disease pathogenesis are needed to correct these circumstances and provide clues for correct treatments choice. Thus, identifying appropriate disease markers/indicators for patients is an emerging issue.

## 3 Precision medicine and biomarkers in SSc

Recent advances in -omics technologies over the past several years and the explosive growth in the use of genome-wide gene expression studies to analyze molecular information from individual patients have revolutionized the field of precision medicine (39). The conventional patient management approach for symptom-centered diagnosis and treatment is limited by its focus on a few late or terminal disease manifestations and by its disregard for the underlying pathogenic mechanisms. Previously, the contribution of an individual’s genetic constitution to the development of these symptoms was not routinely considered, and an emphasis on diagnostic criteria for complex human disorders enhanced these diagnostic and therapeutic limitations. It is possible to handle big data on transcription products (including coding and non-coding RNAs) and their corresponding translated protein molecules in large cohorts of patients, rendering integrative analysis of individual genetic variations (37). Such investigations will result in improvements in the precise diagnosis, in addition to a greater understanding of the complex pathogenic mechanisms responsible for comorbidities in the future (40).

With respect to clinical practice in SSc, the majority of SSc patients carry disease-specific autoantibodies, including antibodies against topoisomerase I, centromere, RNA polymerase I/III, Th/To, U3 RNP, U11/U12, Ku, human upstream-binding factor, U1RNP, and PM-Scl (27, 37, 41, 42). Since autoantibodies are disease-specific, the autoantibody profile has been progressively investigated to determine the subtype, diffuse cutaneous SSc (dcSSc) or limited cutaneous SSc (lcSSc), and predict the disease course (43, 44). Nailfold capillaroscopic microvascular patterns in SSc patients was associated with clinical phenotype and distinctive immune cell abnormalities, which might be linked to stratify individual immune cell target therapies (45). Additionally, empowered -omics studies in SSc have substantial potential to make prognostications and prospect responsiveness to antifibrotic and/or immune target therapies (46–51). In these studies, molecular signatures identified fibroproliferative, inflammatory, and other subset of SSc. Fibroproliferative subset will be linked to antifibrotic drug therapy, whereas inflammatory subset indicates immunosuppressive treatments. This is a major progression for treatment of SSc. However, a portion of this movement is possible to progress toward efficient/preferable gene alterations during the already developed therapies for SSc. Here is a substantial risk of enhancement of unnecessary stratification, or gene-editing therapies in stem/germline cells (52). “Mechanical” decision making might recommend “engineering” persistent genetical risk for SSc beyond the current ethics concern. Thus, further progress in this field requires a modern/future-level consensus.

## 4 Treatment perspective

There are many promising inputs from approximately 550 trials of SSc listed on ClinicalTrials.gov (53). Major part of these trials have used, or are using, new biologic agents or kinase inhibitors such as abatacept, tocilizumab, rituximab, inebilizumab, imatinib, dasatinib, fasudil, nintedanib, lysophosphatidic acid receptor 1 blockers, and fresolimumab, an anti-transforming growth factor (TGF) antibody (54). The results of these trials had a major impact on understanding of the pathogenesis of SSc. In this section, we focus on immunosuppressive B cell depletion therapy and antifibrotic Imatinib therapies, which show some progression of a precision medicine approach for skin sclerosis of SSc.

### 4.1 B cell depletion

Recently, several studies on the role of B cells in autoimmune diseases have been published. B cells contribute to immune responses not only through antibody production but also through antigen presentation, regulating T-cell activation and production of various cytokines. Therefore, B cell-targeted clinical trials for autoimmune disorders are increasing (55). Most patients with SSc have disease-specific autoantibodies, hypergammaglobulinemia, and abnormality in B cell (56, 57). Anti-CD20 monoclonal antibodies deplete most B lymphocytes and abnormal B-cell lymphomas via antibody-dependent cellular cytotoxicity (ADCC) and complement-dependent cytotoxicity (CDC)-dependent mechanisms (58–60). In a murine model of SSc, B-cell depletion with an anti-CD20 monoclonal antibody ameliorated the development of skin sclerosis and autoantibody production (61). Rituximab, a chimeric monoclonal antibody against CD20, was efficacious for both skin sclerosis and ILD in SSc in case reports (62, 63), open-label trials (64–66) and small-scale RCTs (67, 68). In a case–control study of 25 patients with severe SSc (MRSS >16), rituximab treatment significantly improved skin sclerosis (mean ± SEM MRSS, from 26.6 ± 1.4 to 20.3 ± 1.8; P = 0.0001) after 6 months (range, 5–9) (66). Additionally, nine SSc patients with ILD and rituximab preserved %FVC (mean ± SEM %FVC, from 60.6 ± 2.4 to 61.3 ± 4.1; P = 0.5) (66). Consistently, rituximab significantly reduced MRSS while retaining pulmonary function at 12 months in an open-label, uncontrolled study of 20 patients with SSc (64). Therefore, B-cell depletion therapy may be beneficial for patients with SSc with severe skin sclerosis and ILD (67, 69). In addition, drugs for other B cell targets, such as CD19, are also under investigation because these efficiently improve autoantibody reactions in autoimmune disorders (70–73). Interestingly, multiple CD19-target approaches are participating in clinical trials in addition to anti-CD19 antibodies, since CD19 is relatively fragile on B cell surface, in contrast to stable CD20, during ADCC.

### 4.2 Imatinib mesylate

IM is a tyrosine kinase inhibitor that inhibits c-Abl, an important downstream signaling molecule of TGF-beta, and the platelet-derived growth factor signaling pathway, which stimulates collagen production (74). In a mouse model of bleomycin-induced lung fibrosis, IM was shown to prevent TGF-beta-induced fibroblast proliferation and ECM gene expression, resulting in the suppression of lung fibrosis (75). Moreover, in mouse models of SSc, IM inhibited both TGF-beta and platelet-derived growth factor signaling, leading to suppression of skin fibrosis (76, 77). Although IM may be a promising option for the treatment of SSc, based on the results of animal studies, its efficacy remains controversial in clinical trials. In a phase I/IIa, open-label, pilot trial with 20 patients with SSc, IM improved both %FVC and MRSS (1.74% and 3.9 points, respectively) at 12 months, although 12 of 20 patients completed the study mainly because of the adverse effects of IM (78). In a phase II pilot study, 30 SSc patients with ILD resistant to cyclophosphamide were treated with IM for 6 months (79); 19 of these 26 patients (73%) showed stabilized pulmonary function. In contrast, in a phase II RCT, included 28 patients with SSc, did not show significant improvement in skin sclerosis at 6 months (23). Further studies are needed to validate the safety and efficacy of IM with definitive inclusion subsets of SSc (80).

## 5 Perspective

**Figure 1** shows a schematic of the pathological pathways involved in SSc. Various endogenous factors in cooperation with exogenous triggers establish heterogeneous phenotype in SSc. The pathogenetic stream narrowed weekly during the establishment of phenotypic characteristics (**Figure 1B**). The results from clinical practice indicate that antifibrotic drugs, including IM, Tocilizumab, are effective for specific patients with SSc, although these were not validated in randomized trials in patients with SSc (81–83). This indicates that narrow-target therapy partially abrogates wide and complex pathways to SSc. Other spiral vectors bypass the blocking point and sometimes amplify/enhance the continuous development of SSc phenotypes (**Figure 1B**). By contrast, IL-23 target therapy is a focus point in the treatment of psoriasis (**Figure 1D**). Tumor necrosis factor, locating near the focus point, therapy sometimes cause paradoxical enhanced reactions in psoriasis (84). Great effort will tighten and clear the center of the motif in SSc, as well. **Figures 1A–D** directed-clearance looks like tightening the center cord of the motif “Tuzumi” (**Figure 1G**). We named this as adjustment cord theory.

Biomarker studies focusing on autoantibody profiles and other candidates may classify/hierarchize patients with SSc to select an efficient patient-entry design in clinical trials (**Figure 1F**). Further characterization of the key genomic and immune pathways involved will help to identify novel disease mechanisms and therapeutic target patients for precision medicine initiatives in SSc. For example, a higher IFN-inducible protein (IP10) level in SSc-ILD can predict the response to MMF or CYC (85, 86). CD19-positive B cell counts were associated with anti-CD20 Ab effectiveness (87) These initiated precision medicine for SSc (**Figure 1F**).

Alternatively, focusing on the final fibrotic process is empowered to change mortality or quality of life, since the final step for phenotypic fibrosis seems to be crucial for mortality and quality of life in most patients with SSc (**Figure 1E**). Fibrosis is caused by the overproduction of collagen and other ECM components by activated fibroblasts and α-smooth muscle actin positive myofibroblasts (88). Multiple physiological mechanisms regulate fibroblast activation to prevent excessive tissue remodeling and fibrosis (89). These complex reactions intensify cellular collagen overproduction, which has a long history of focus as a critical therapeutic target for SSc. Accordingly, patients belonging to the fibroproliferative intrinsic subset may respond to the antifibrotic drug IM (49). A better understanding of this stratification and precision medicine will lead to safer and more effective development of new antifibrotic drugs for SSc. However, this approach using a narrow target reagent still carries a substantial risk of adverse/paradox enhancement reactions, since it is impossible to find a focus point in SSc (**Figure 1E**).

SSc is a rare and diverse condition. The relevant environmental associations and the genes responsible for SSc are still unknown. Nonetheless, several potential drugs are currently available for specific patients, if not all SSc. Therefore, we propose a drug selection system using *in vitro* fibroblast culture (90). To select the potential drugs for a patient, we treated SSc skin fibroblast cell lines using four candidate substances/drugs affecting SSc related pathophysiologic processes.

**Figure 2:**
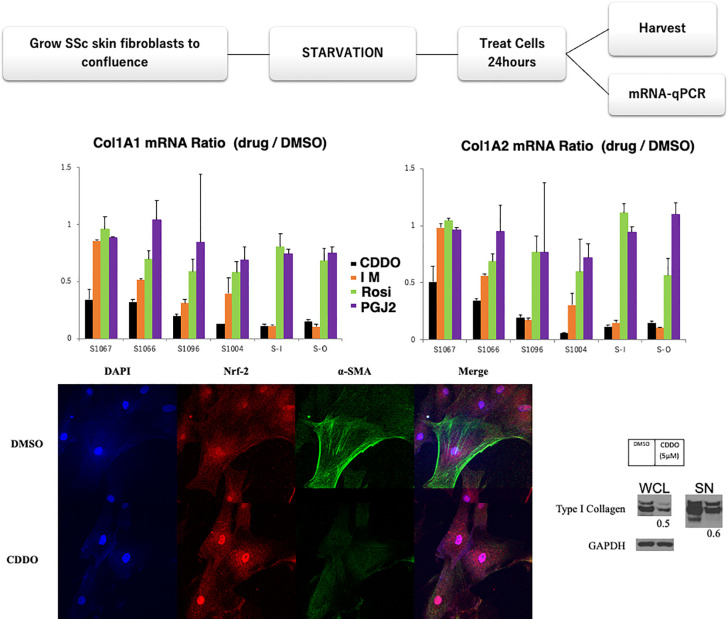
Personalized drug testing model in SSc. Antifibrotic drugs were selected based on the results of preclinical functional/treatment-based assessments. Six SSc skin fibroblast cell lines were treated with the anti-fibrotic agents bardoxolone (CDDO, 5µM), imatinib (IM,10µM), rosiglitazone (Rosi,10µM), PGI2 (10µM), or DMSO (as control). Starved fibroblasts were incubated with each treatment for 24 h. Purified mRNA from whole-cell lysates (WCL) was analyzed using real-time qPCR. Results are expressed as the ratio of Type I and II collagen mRNA levels relative to those from fibroblasts treated with DMSO. The anti-fibrotic drug responses were different between patients’ fibroblasts. Lower panel: CDDO treatment downregulated alpha-SMA expression in SSc skin fibroblasts with nuclear translocation of Nrf2, a key transcription factor that regulates antioxidant activity. Confluent SSc fibroblasts were incubated with DMSO (control) or 5µM CDDO for 24 h. Cells were fixed and stained with antibody for Nrf2 at 1:100 dilution (Santa Cruz) or alpha-SMA Ab (Sigma). Lower right panel: CDDO treatment downregulated type I collagen expression in SSc skin fibroblasts. Starved fibroblasts were incubated with CDDO for 24 h. WCL and culture supernatants were analyzed by Western analysis. Bands were quantitated by densitometry. Relative levels normalized with GAPDH are shown below the images. Representative images.

Six SSc skin fibroblast cell lines were treated with four low molecular antifibrotic compounds, including bardoxolone (Nrf2, a key transcription factor that regulates antioxidant activity, inducer: CDDO, 5mM), imatinib (a tyrosine kinase inhibitor: IM,10mM), rosiglitazone (PPARgamma agonist: Rosi,10mM), PGJ2 (PPARgamma agonist,10mM), or DMSO (control) for 24 h. The details of the clinical data of these patients are described in patient background (**Table 1**). The starved fibroblasts were incubated with each reagent for 24 h. Purified mRNA from whole-cell lysates (WCL) was analyzed using real-time qPCR. Results are expressed as the ratio of Type I or II collagen mRNA levels relative to the control fibroblasts (**Figure 2**). As observed from the graphs in **Figure 2** for two patients with CDDO has similar effect for IM (SSc-1096, SSc-I). CDDO for other three patients (SSc-1067, SSc-1066, SSc-1004) were more efficient than Im in collagen inhibition, while Rosi and PGJ2 both had very little effect in all patient cell cultures. Thus, CDDO improved all patients’ collagen synthesis(**Figure 2**), suggesting CDDO as first line treatment. Further, these results suggest that using IM for some patients (SSc-O) could be more efficient than CDDO. IM showed limited effect on SSc-1067 and SSc-1066 collagen production (**Figure 2**): SSc1066 treated by Im in past however currently treated by MTX, because IM showed limited merit for SSc 1066 (**Table 1**). In addition, no benefit was observed from Rosi or PGJ2, which may link to negative results of clinical trials with PPARgamma agonists. We could not find any association between clinical feature and current *in vitro* results (**Table 1** and **Figure 2**) concerning the effect of CDDO and IM.

**Table 1:** Clinical characteristics of patients with SSc.

Study Code	Age	Early/Late (<2 yrs for early)	mRTSS	Autoantibody	Current Treatment	Past Treatment
SSc-1067	56-60	Late	34	ANA (non-specific)	MMF	Penicillamine
SSc-1066	21-25	Early	15	Topoisomerase-I	MTX	IM
SSc-1096	26-30	Late	5	U1RNP	–	–
SSc-1004	26-30	Late	26	ANA (non-specific)	–	MMF
SSc-I	71-75	Early	19	ANA (non-specific)	–	–
SSc-O	31-35	Late	unknown	RNA polymerase I/III+II	–	–

dcSSc, diffuse cutaneous SSc; lcSSc, limited cutaneous SSc; ANA, antinuclear antibodies; MTX, methotrexate; MMF. MMF, mycophenolate mofetil.

The anti-fibrotic effect of CDDO was confirmed by immunocytochemistry and western blotting (**Figure 2** lower panels), according to the protocol described elsewhere (89). CDDO treatment downregulated alpha-SMA expression in SSc skin fibroblasts with nuclear translocation of Nrf2. This process represents an optimized environment for dermal fibroblasts to normalize collagen production. Hence, this personalized drug testing system has a potential to provide additional precision medicine for SSc. We can cultivate and investigate these tests from every patient within two months from skin biopsy. This enables physicians to know the results before starting the fundamental treatment for the patients. Further, this approach could respond the issue about other organ fibrosis, including lung. However, enrichment of the number of patients with medical outcomes and many candidate anti-fibrotic drugs will be required. In addition, other outcome indicators, including and fibronectin gene and protein expression, are also needed for this experimental design.

Classification based on drug response is likely to form the foundation for better therapeutic strategies in SSc with Fibrotic phenotype (**Figure 1F**). In clinical practice, we cannot clear-cut fibrotic phenotype from inflammatory phenotype, since fibrotic process is interacting with inflammatory process (91). However, personalized drug testing system could lead to improvements in the design of appropriately stratified clinical trials to determine the effects and prediction of targeted therapies.

The precision medicine in SSc is only beginning. The main hurdles to move forward include the complexity of disease, requirement for ethical consensus, and imbalance to access medical resources. The globalization of world enables to share, compare, and analyze datasets of patient for this rare disease. Thus, recent circumstances continue to develop personalized treatments and precision medicine for SSc patients, providing promising outlook.

## Data availability statement

The raw data supporting the conclusions of this article will be made available by the authors, without undue reservation.

## Ethics statement

The studies involving humans were approved by Northwestern University Institutional Review Board for Human Studies. The studies were conducted in accordance with the local legislation and institutional requirements. The participants provided their written informed consent to participate in this study.

## Author contributions

KK: Conceptualization, Data curation, Formal Analysis, Funding acquisition, Investigation, Methodology, Project administration, Resources, Software, Supervision, Validation, Visualization, Writing – original draft, Writing – review & editing. KY: Writing – review & editing, Methodology. J-DB: Methodology, Writing – review & editing. AY: Writing – review & editing. MH: Writing – review & editing. JV: Conceptualization, Data curation, Funding acquisition, Investigation, Methodology, Project administration, Resources, Supervision, Writing – original draft. KT: Methodology, Writing – original draft. TM: Resources, Supervision, Writing – review & editing.
